# Modification of Bacterial Cellulose Biofilms with Xylan Polyelectrolytes

**DOI:** 10.3390/bioengineering4040093

**Published:** 2017-11-28

**Authors:** Sara M. Santos, José M. Carbajo, Nuria Gómez, Miguel Ladero, Juan C. Villar

**Affiliations:** 1 Laboratory of Cellulose and Paper, INIA, Forest Research Center, Ctra. De la Coruña km 7.5, 28040 Madrid, Spain; chema@inia.es (J.M.C.); nuria@inia.es (N.G.); villar@inia.es (J.C.V.); 2 Department of Chemical Engineering and Materials, Universidad Complutense de Madrid, Avda. Complutense s/n, 28040 Madrid, Spain; mladero@quim.ucm.es

**Keywords:** bacterial cellulose, xylan polyelectrolytes, nanocomposites

## Abstract

The effect of the addition of two [4-butyltrimethylammonium]-xylan chloride polyelectrolytes (BTMAXs) on bacterial cellulose (BC) was evaluated. The first strategy was to add the polyelectrolytes to the culture medium together with a cell suspension of the bacterium. After one week of cultivation, the films were collected and purified. The second approach consisted of obtaining a purified and homogenized BC, to which the polyelectrolytes were added subsequently. The films were characterized in terms of tear and burst indexes, optical properties, surface free energy, static contact angle, Gurley porosity, SEM, X-ray diffraction and AFM. Although there are small differences in mechanical and optical properties between the nanocomposites and control films, the films obtained by BC synthesis in the presence of BTMAXs were remarkably less opaque, rougher, and had a much lower specular gloss. The surface free energy depends on the BTMAXs addition method. The crystallinity of the composites is lower than that of the control material, with a higher reduction of this parameter in the composites obtained by adding the BTMAXs to the culture medium. In view of these results, it can be concluded that BC–BTMAX composites are a promising new material, for example, for paper restoration.

## 1. Introduction

Bacterial cellulose (BC) is produced by certain bacterial species. BC consists of elementary fibrils of pure cellulose, free of lignin and hemicellulose, and it is considered a natural nanocellulose [1,2]. These elementary fibrils make up a flat, ribbon like microfibril, which are branched together in the BC films, providing high mechanical strength [3]. BC shows high crystallinity, high water absorption capacity, and high degree of polymerization [4]. These properties, along with its biocompatibility, make it an attractive candidate for a big range of applications in various fields, receiving much attention as potential materials associated with biomedical and biotechnology applications [2].

BC is an excellent matrix for the preparation of composites. The use of cellulosic materials in polymer composites has been growing over the past, due to advantages such as low density, renewability, and biodegradability [5]. BC may be used in high quality special applications of bio-based composites, because of the small fibril dimensions that enable direct contact between cellulose and polymers, allowing for a large contact surface, and thus, excellent adhesion. BC composites showed considerably improved properties, leading to additional applications in the medical and other industrial fields [6].

BC permits two ways of composites forming: by in situ BC modification, adding composite partners to the culture medium, or by processing BC previously synthesized [7]. In the literature, different compounds have been added to study their influence on yield, morphology, and crystalline constituents of BC, including agar [8], sodium alginate [9], carboxymethylcellulose [10,11], pectin [9], carbon nanotubes [12], polyacrylamide [13], xylan [14], xyloglucan [15], acetyl glucomannan [16], lignosulfonate [17], and microcrystalline cellulose [18].

It has been proven that xylans interact with cellulose, and are irreversibly absorbed onto cellulosic surfaces [19]. Adsorption of the pre-isolated xylans has been shown to improve pulp properties, such as tensile strength, beatability and resistance to hornification [20]. However, it is well known that only low amounts of xylan can be adsorbed in comparison with xylan derivatives [21]. Despite the use of xylans for cellulose fiber modification, these polymers can be transformed into new polymers with promising properties by chemical modification [22]. The cationic ammonium xylan derivatives are described to enhance the tensile modulus of pulp, compared to the untreated pulp [23]. Vega et al. [24] produced and characterized [4-butyltrimethylammonium]-xylan chloride polyelectrolytes. They observed, by using time-of-flight secondary ion mass spectrometry (ToF-SIMS), that BTMAXs were adsorbed onto the surface of cellulosic fibers of bleached pine kraft pulp. They concluded that a positive polyelectrolyte prepared from extracted xylan could be used as a modifying fiber surface agent. Therefore, certain improvements on BC properties are expected by including BTMAXs during its production.

In the present study, we have produced BC/xylan polyelectrolytes nanocomposites by incorporating xylan polyelectrolytes by two different approaches. In the first process, xylan polyelectrolytes were incorporated in BC hydrogels by adding them to the medium for *Gluconacetobacter sucrofermentans*. The second process was based on the penetration and adsorption of the xylan polyelectrolytes in a BC hydrogel that was previously produced and homogenized. The obtained BC composites were characterized in terms of tear and burst indexes, optical properties, static and dynamic contact angles, Gurley porosity, SEM, X-ray diffraction, and AFM.

## 2. Materials and Methods

### 2.1. Microorganism

*Gluconacetobacter sucrofermentans* CECT 7291 was obtained from the Spanish Type Culture Collection (CECT, Valencia, Spain). For maintenance, it was subcultured periodically in HS medium [25]. *G. sucrofermentans* was grown in HS solid medium placed in Petri dishes for 6 days, in order to obtain the suspension of bacterial cells to be utilized in further experiments. Five hundred milliliter Erlenmeyer flasks containing 100 mL of liquid HS medium were inoculated with biomass from these dishes, and cultivated in static conditions for 4 days. Subsequently, the pellicles formed were cut in small pieces (about 1 cm × 1 cm) in sterile conditions, and shaken with the liquid medium at 700 rpm for 30 min. The suspension obtained was filtered through gauze, centrifuged at 4000 rpm for 10 min, and after removing the supernatant, the pellet was washed with Ringer’s solution (NaCl, 2.5 g/L; KCl, 0.105 g/L; CaCl_2_·2H_2_O, 0.120 g/L; and NaHCO_3_, 0.05 g/L). The solid phase was centrifuged again in the same conditions, and the final pellets were re-suspended in a small volume of Ringer’s solution. The optical density of the suspension at a wavelength of 600 nm was adjusted to 0.59–0.64 (McFarland standards 3-4) diluting when needed with Ringer’s solution. Of this final solution, 250 µL was used to inoculate 100 mL of medium.

### 2.2. Films Production and Purification

The objective is to obtain BC films modified with xylan derivatives by two different strategies, one based on the addition of the polymers to the culture medium during the synthesis of BC (s), and a second one adding the polymers to the BC already generated and homogenized (h).

Polymers, named PS5 and PS6, are two xylan derivatives modified with 4-[*N*,*N*,*N*-trimethylammonium]butyrate chloride (XTMAB), with different degrees of substitution (0.13 for PS5 and 0.58 for PS6). These polyelectrolytes were a kind gift from the research group of P. Fardim at Åbo Akademi in Turku (Finland). According to Vega et al. [24], the molecular weight of the repeating unit of the polymer is 200 g/mol, and the saturation of cellulose with the polymer is reached with 70 μmol of functional groups per gram of pulp (BC in this case). In order to maximize xylan–BC interactions, xylan derivatives were added so as to reach the saturation point (27 mg of PS5/BC sheet and 6 mg of PS6/BC sheet).

Culture medium employed for BC production in both cases, (s) and (h), was a modified HS medium (fructose, 20 g/L; yeast extract, 5 g/L; corn steep liquor, 5 g/L; Na_2_HPO_4_, 2.7 g/L; and citric acid, 1.15 g/L). In all cases, 100 mL of liquid medium were added to 150 mm Petri dishes, inoculated with the suspension described above, and cultivated at 30 °C under static conditions. As it has been described previously [26], in one week under these conditions, BC films of approximately 0.25 g dry weight are obtained.

For BC composites generated during BC synthesis (s), polymers were added to the culture medium as inoculation was performed (BC_PS5_s_; BC_PS6_s_). Also, control films without polymer have been generated (BC_C_s_). All sheets were collected after 7 days of culture, purified by boiling them for 1 h at 90 °C in 1% NaOH, and washing them afterwards, thoroughly with distilled water. They were dried by filtration on a Büchner funnel and air-dried afterwards.

For BC composites from the BC previously homogenized (h), BC films have been obtained in the same way as control ones. After washing them as described previously, they were gelled using a Panda Plus 2000 homogenizer (GEA Niro Soavi, Parma, Italy), treating them five consecutive times at a pressure of 600 bar, with a subsequent consistency adjustment at 1%. After that, xylan derivatives have been added, adjusting again the consistency at 0.5%. Appropriate amounts of gel were placed in Petri dishes and allowed to dry in order to obtain layers (BC_PS5_h_; BC_PS6_h_). Control layers (BC_C_h_) were prepared in the same way, but avoiding addition of xylan derivatives.

### 2.3. X-ray Diffraction (XRD)

BC crystallinity was studied by XRD using a multipurpose PAN analytical diffractometer (PANalytical, Almelo, Netherlands), model X’PertMPD. This diffractometer is equipped with a copper X-ray tube and two goniometers with vertical configuration th-2th and Bragg–Brentano optic. One of the goniometers has a multipurpose sample support, which can hold large samples as heavy as 1 kg and measuring up to 10 cm × 10 cm × 10 cm. The supporting platform of the second goniometer is a sample spinner fitted with an automatic sampler with 21 positions. The diffractometer is used in phase analysis and, in this study, the angular range between 2*θ* = 5°–40° was studied. The crystallinity indexes for the BC samples have been calculated in each case using the Equation (1) [27]:

(1) $$Crystallinity\;\left(\%\right)=\;\frac{I_{200}-I_{2\theta =18}}{I_{200}}\;\times 100$$

### 2.4. Contact Angle Measurements and Surface Free Energy Determination

The samples’ wettability was studied by means of contact angle measurements in air (α), using distilled water. This technique has been also used to obtain the samples surface free energy, since the contact angle measurement, using liquids whose surface tension is known, is an indirect method to analyze this parameter, being fast, simple, and based on easy-to-use equations. Several studies can be found in the open literature concerning the fundamentals of this method [28,29,30].

The measurements were performed in a DataPhysics Instrument OCA 15 plus, running on SCA 20/21 software (DataPhysics Instruments GmbH, Filderstand, Germany) and using the sessile drop method. A CCD camera takes the images of the initial resting drop immediately after the drop impacts on the paper surface. The corresponding contact angle is calculated after fitting the drop contour line numerically, using the Young–Laplace method. In this study, a 15 drop test was made for distilled water, applying a drop volume of 2 µL.

The surface free energy (*γ*) was calculated by the OWRK (Owens, Wendt, Rabel and Kaelble) method, which considers the interfacial tension as a function of the dispersive (γ_d_) and polar (γ_p_) interactions [30]. In this study, the probe liquids were *n*-hexane, ethylene glycol, 1,2-propanediol, formamide, and distilled water, using the 15 drop test, and 5 µL for each one.

The influence of surface topography in the contact angle has been rather reported by several authors, who recommended using the Wenzel´s roughness correction [31,32,33]. The Wenzel Equation (2) establishes that the relationship between the measured contact angle (α_m_) and the corrected angle in an ideal flat surface (α_c_) may be written as follows:

(2) $$\cos\alpha_{m}=r\;\times \cos\alpha_{c}$$

where *r* is the topographical correction factor Equation (3) obtained as:

(3) $$r=1+\;\frac{Sdr}{100}$$

where *Sdr* is the developed interfacial area ratio provided by atomic force microscopy (AFM). The Wenzel’s correction was carried out for each contact angle measured in this study.

### 2.5. Scanning Electron Microscopy (SEM)

The morphology of the BC layers was studied by SEM microscopy using a JEOL JSM 6335F (JEOL, Peabody, MA, USA) at 1 kV (with maximum resolution of 5 nm) to avoid energetic degradation of the samples during SEM observation. Samples have been previously cryo-fractured after immersing them into liquid nitrogen, and metalized with gold during 3 min and stored for 16–18 h at 50° in a vacuum stove (20 mmHg) before proceeding with SEM observations. This latter treatment led to total dryness of the samples.

### 2.6. Atomic Force Microscopy (AFM)

The BC derivatives were characterized by means of AFM imaging using a Nanoscope IIIa Bruker microscope (Bruker, Billerica, MA, USA). The images were scanned in the AFM in tapping mode in air using 2 nm radio cantilevers (TESP-SS Bruker, Bruker, Billerica, MA, USA). The drive frequency of the cantilever was approximately 0.4–0.5 Hz. The images were 10 × 10 μm. Scan size 10 µm, tip velocity 8 µm/s, 256 lines, aspect ratio: 1:1, scan angle 0, Z range 1500 nm. Except for flattening, no image processing was conducted. The surface roughness was evaluated with the root mean square average of height deviation taken from the mean image data plane (Rq).

### 2.7. Mechanical and Optical Properties

The following mechanical properties were determined: basic weight (ISO 536:2012), burst strength (ISO 2759:2001), and tear strength (ISO 1974:2012). A Color Touch reflectometer (Datacolor^®^, Lawrenceville, NJ, USA) was used to assess the optical properties: ISO brightness (ISO 2470-1:2009), opacity (ISO 2471:2008), and yellowness index (SCAN-G 5:03). Yellowness is defined as the attribute by which an object color is judged to depart from a preferred white toward yellow.

The specular gloss was determined using an angle of incidence of 75° (ISO 8254-1:1999). To measure the air permeance of papers, a Gurley porosimeter was used (ISO 5636-5:2013).

## 3. Results and Discussion

### 3.1. X-ray Diffraction

**Figure 1:**
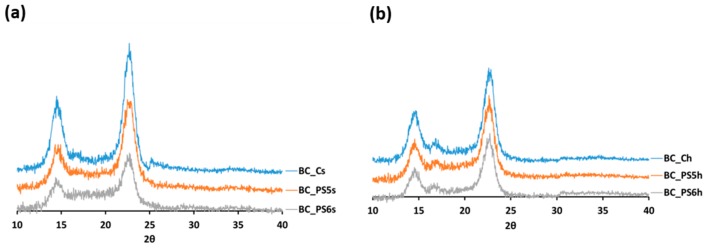
X-Ray diffraction patterns of (**a**) samples obtained from non-homogenized bacterial cellulose (BC), and (**b**) samples obtained from homogenized BC.

X-ray diffraction was used to study the structure of the obtained materials (Figure 1). Each of them has two diffraction dominant peaks, one disposed between 14° and 15°, and the other between 22° and 24°. Each of the peaks presents the two crystalline phases, Iα and Iβ. According to Barud et al. [34], who investigated membranes of BC, the first peak represents the projection of the planes (1 0 0) of fraction Iα and (1 1 0 and 0 1 0) of fraction Iβ, and the second peak represents the projection of the planes (1 1 0) Iα and (2 0 0) I Iβ.

The obtained diffractograms suggest that the addition of BTMAXs did not alter BC crystalline morphology in any case, which is consistent with the results obtained by Huang et al. [35]. A decrease in peak intensity can be observed around 2*θ* = 17° in the samples from the generation with the BTMAXs in culture medium, compared with their control (Figure 1a). This behavior was also observed by Khan et al. [36]. This peak is also evident in the samples from the homogenized BC (Figure 1b), regardless of whether they have polyelectrolytes or not.

The crystallinity indexes of the materials are shown on Table 1. It can be observed that materials made only with BC are more crystalline than the materials with xylan polyelectrolytes, regardless of the method of production. The amorphous nature of BTMAXs could be the cause of decreased crystallinity, being these results in agreement with previous studies showing a reduction in crystallinity due to the presence of additives [36,37].

As it can be observed, when BTMAXs are added to the culture medium, the crystallinity decrement is more pronounced than if the addition of the polymer is done to the homogenized BC. The reason may be found by Huang et al. [35], who observed that hydroxypropylmethyl cellulose or carboxymethyl cellulose do not stick regularly on individual microfibrils, preventing the aggregation of microfibrils and ribbon formation during fermentation. Hirai et al. [11] concluded that the effect of polymer additives on the formation of microfibrils of BC depends on their degrees of polymerization or substitution, which can explain the differences in the crystallinity between PS5 and PS6.

When the addition of the polyelectrolytes is carried out after the homogenization of BC, the decrease in crystallinity is slight. This was also observed when chitosan (Ch) is included after the BC formation: the intermolecular hydrogen bonding interaction between the BC and Ch molecules promotes a slight decrease in the crystallinity index of the BC_C_h_ composites, compared to pure BC [38].

Yano et al. [39] used two processes to prepare nanocomposites of BC filled with silica particles. In one of them, *G. xylinus* was incubated in medium containing silica particles, which disturbs the formation of ribbon-shaped fibrils and affects the preferential orientation of the (110) plane, modifying the crystallinity. In the other process, the BC hydrogel was immersed in different concentrations of silica solutions, allowing silica particles to diffuse into the BC hydrogel and lodge in the spaces between the ribbon-shaped fibrils, hardly affecting the crystallinity.

### 3.2. Contact Angle Measurements

The surface free energy and polar/dispersive components of samples are presented in Table 2. As it can be seen, both biofilm controls showed similar values of surface free energy and its components, as expected.

The use of BTMAXs to obtain composites led to a change in the surface free energy and its components, but the behavior depends on the way of obtaining the biofilms.

When the BTMAXs were added to culture medium, the resulting γ values were smaller than the control one, whereas the γ values were increased when blending them with the homogenized BC. These data are in agreement with those by Lopes et al. [40], who suggested that hyaluronic acid (HA) added to the culture medium are retained inside the membrane. Therefore, during the BC synthesis, the free hydroxyl groups of the newly formed BC fibrils orientate toward the HA, becoming no longer available to occupy the surface, and lowering the surface hydrophilicity of the material.

On the contrary, when BTMAXs are mixed with the homogenized BC, there could be polar groups on the surface of the composite, from both the BC and the polyelectrolytes. This would increase their surface free energy. The more hydrophilic character of these surfaces is in accordance with Harnett et al. [41], who observed that low surface energy values correspond to surfaces with high hydrophobic character. This is in accordance with Vega et al. [24], who observed that the BTMAXs were evenly adsorbed onto the surface of pulp fibers, following Langmuir model.

**Figure 2:**
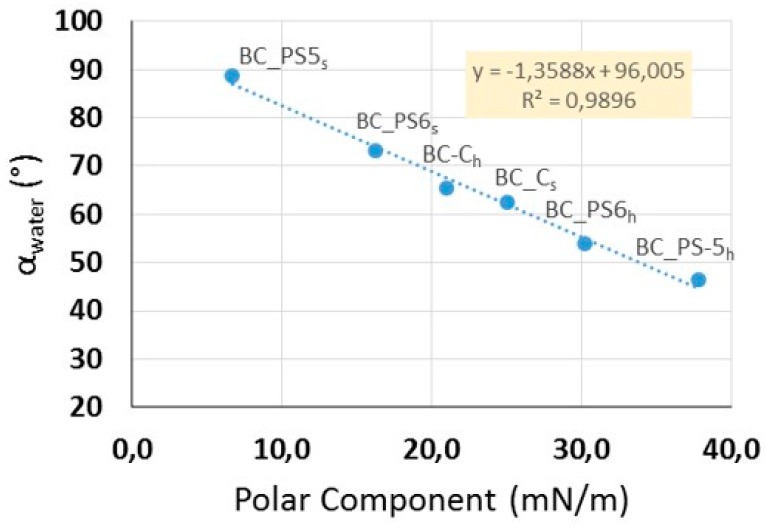
Relation between the polar component of surface free energy of the samples and their static contact angles obtained with water.

The composites showed quite similar values of dispersive component (γ_d_), ranging from 10.0 mN/m to 18.2 mN/m, whereas variability in the polar component (γ_p_) was greater, from 6.7 mN/m to 37.8 mN/m. This polar component is related to the cellulose wettability with water. Thus, material with a high γ_p_ has greater affinity for water, resulting in low static contact angles [31,42,43]. Figure 2 shows the relationship between polar component and static contact angle with water (α_w_). It can be observed that α_w_ decreased when the polar component of samples increased. The regression analysis between these two component reveals a significant correlation between both parameters at a 95% confidence level (*r* = 0.99), confirming that the static contact angle between water and material increases with decreasing γ_p_. This finding may provide an important understanding about how to modify the BC to obtain hydrophobic surfaces.

### 3.3. Scanning Electron Microscopy

**Figure 3:**
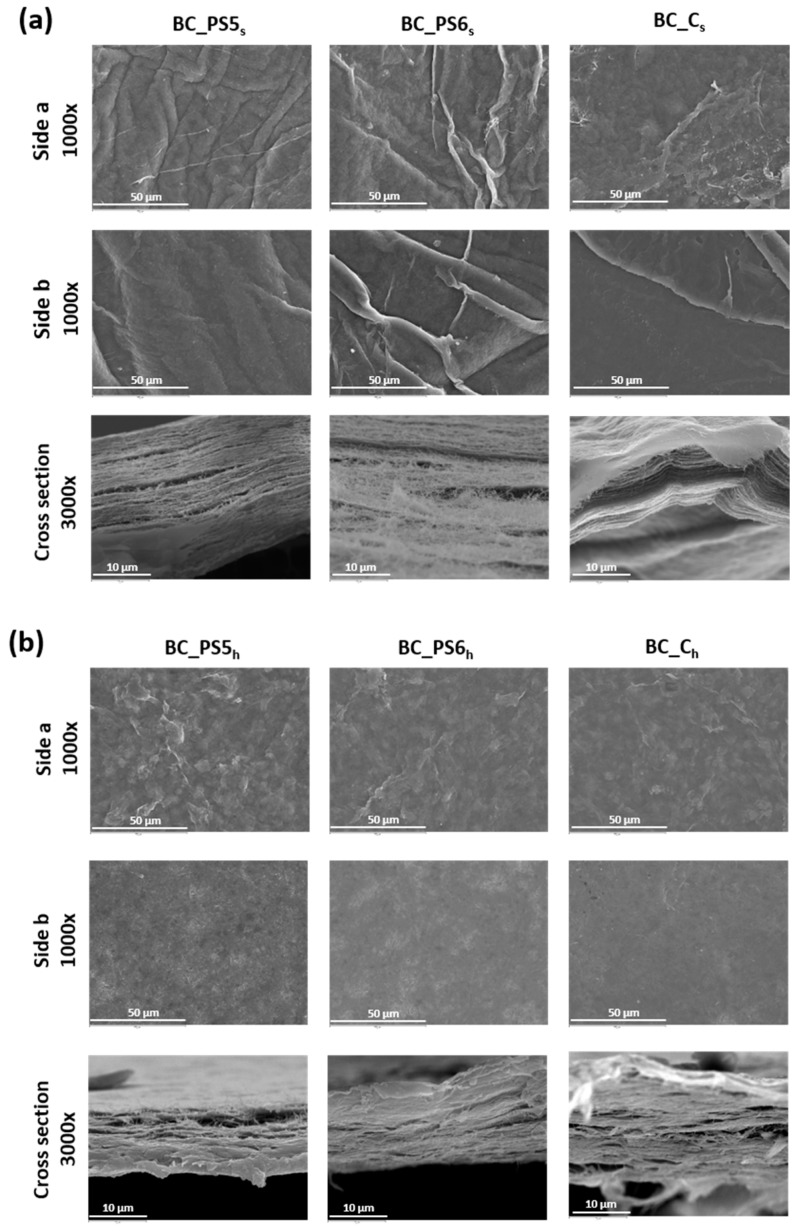
SEM images at different magnifications of samples. (**a**) Samples obtained from non-homogenized BC. (**b**) Samples obtained from homogenized BC.

The morphology of BC dried layers has been studied by SEM microscopy (Figure 3). This technique allows obtaining images of the surface and the cross sections of the samples, so evident qualitative differences between pure BC films and those of nanocomposites can be appreciated.

SEM microscopic images of samples obtained from adding polyelectrolytes to the culture medium (Figure 3a) reveal that the surface of the films depends on their composition. The presence of BTMAX promotes a rougher surface, which supports the specular gloss results (Table 4).

**Figure 4:**
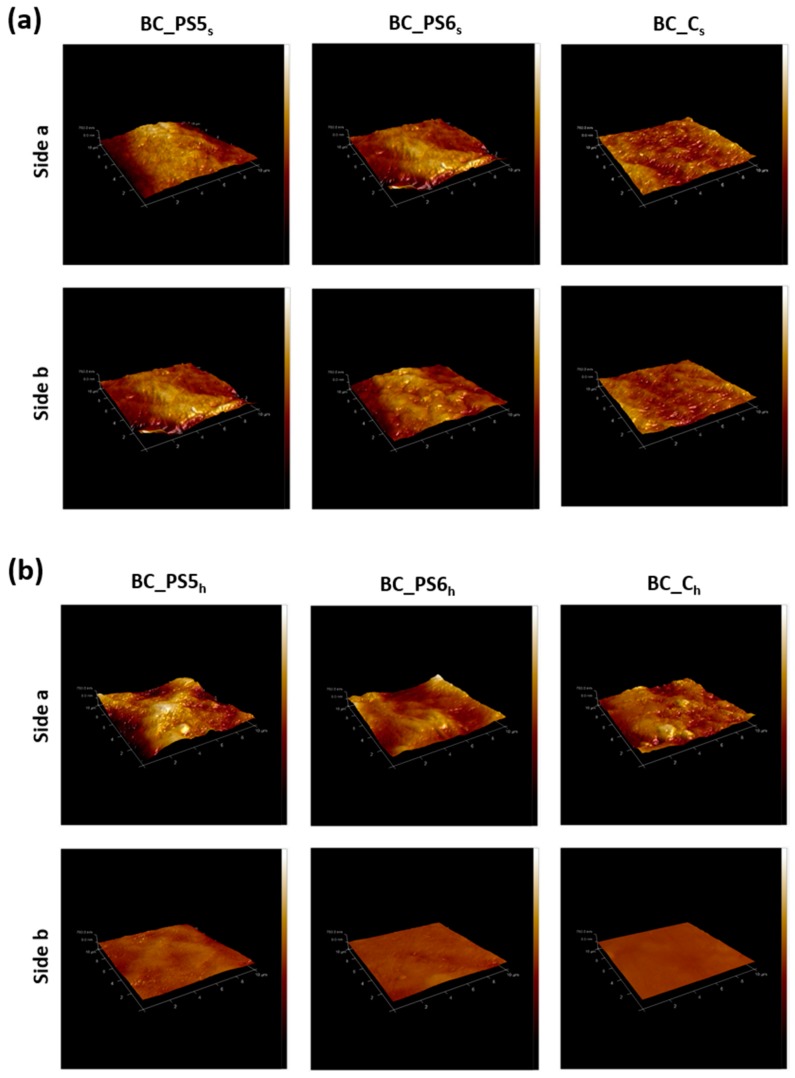
AFM images of samples. (**a**) Samples obtained from non-homogenized BC. (**b**) Samples obtained from homogenized BC. Size of the images: 10 µm × 10 µm × 750 nm.

When the BTMAXs were added to the BC previously homogenized (Figure 3b), the resulting gels were placed in Petri dishes, and allowed to air dry. This resulted in that the faces of the composites that were in contact with the Petri dishes (face b) are clearly smoother. This is confirmed by AFM data and images (Figure 4) and, as will be seen later, by greater gloss values (Table 4). In this case, SEM micrographs of the samples from gel (Figure 3b) allowed us to observe that there are no differences on the surfaces between the added BTMAXs. This is in accordance with data obtained from surface free energy (Table 2), as it was mentioned before.

It was also observed the morphology of cryo-fractured samples, corresponding to the cross sections. The structures of pure BC and nanocomposites were formed by a group of thin sheets, forming a pile. The fact that pure BC and nanocomposites have similar morphology at these scales, confirm that the blending of the BTMAX with BC took place on the nano scale. SEM micrographs corresponding to the transversal sections of the sheets reveal that the process in which the BTMAXs are added to the homogenized BC promotes a more compact structure, if compared to other studied samples.

### 3.4. Atomic Force Microscopy

AFM was used for studying surface roughness at the nanoscale. The obtained values for Rq are shown in Table 3. It was observed that addition of the BTMAX to the culture medium increases the Rq, having rougher surface when compared to the other samples. When the BTMAXs were added to the homogenized BC, an increment is also observed, but in this case, the increase in side b is less prominent. These results are in agreement with the data obtained in surface free energy determinations, SEM microimages, and gloss values.

The qualitative differences between the morphologies of the pure BC samples and those of the nanocomposites can be seen in the AFM images (Figure 4). The images confirm the results obtained by the SEM, the surface free energy measurements, and the gloss values. The polyelectrolytes added to the culture medium (Figure 4a) promote rougher surface formation. On the other hand, the main difference accompanied by polyelectrolytes addition to the homogenized BC (Figure 4b) was the drying method, as it was discussed above.

### 3.5. Mechanical and Optical Properties

**Figure 5:**
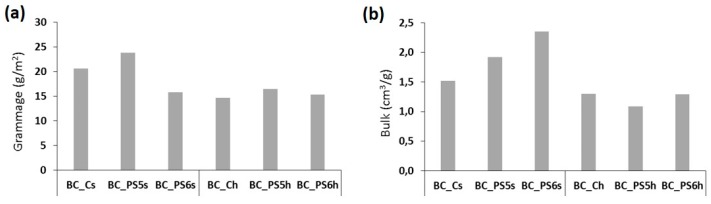
(**a**) Grammage (g/m^2^) and (**b**) bulk (cm^3^/g) of the samples.

The basic weight and the bulk of the samples are shown in Figure 5. According to several studies, the ratio of the biosynthesis of cellulose by *A. xylinum* is changed depending on the culture conditions, e.g., additives and temperature [44,45]. This observation is consistent with our data, shown in Figure 5a. The specific volume or bulk is the inverse of the density, that is, the volume in cm^3^ of 1 g of paper. Bulk provides information on the structure of the sheet, and is related to most of the properties of paper, specially porosity, stiffness, hardness, and strength. Bulk also affects the absorption and the ease of being printed. Bulk can be modified by many factors, as the presence of materials that fill the voids in the sheet, or factors affecting the number of joints between fibers, such as fiber diameter.

When bulk is determined for all samples (Figure 5b), important differences have been found. When composites are made by adding the polyelectrolytes to the homogenized BC, bulk presents similar values in all cases. On the contrary, when the polymers have been added to the culture medium (Figure 5a), resultant samples have higher bulk values, showing a less compact structure, an observation confirmed by SEM images (Figure 3), which showed more compact layers in the case of the samples made from the homogenized BC.

**Figure 6:**
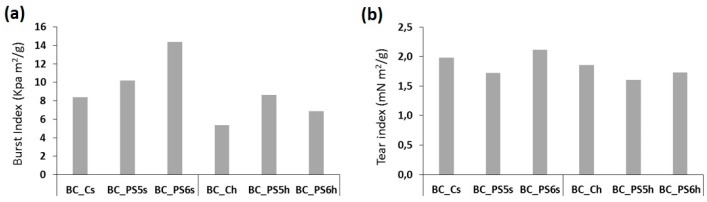
(**a**) Burst index (KPa m^2^/g) and (**b**) tear index (mN m^2^/g) of the samples.

Noticeable results are obtained when mechanical properties (burst and tear) are analyzed, as shown in Figure 6. With both xylan polymers, PS5 and PS6, there is an increment in burst index (Figure 6a), regardless of the strategy followed to obtain the samples (BTMAX addition to culture medium, or addition to the homogenized BC). Similarly, Yano et al. [39] observed an increase in tensile strength by adding silica particles to the BC already formed. Tear index (Figure 6b) remains close to the original value in all cases.

Optical properties of the pure BC and the composites are shown in Table 4. The yellowness is similar in all cases, slightly higher in the case of having added PS5, probably due to the higher amount of polymer. For the same reason, brightness is a little lower when the added polyelectrolyte is PS5. In any case, this variable is a little higher in the case of samples made by adding BTMAXs to homogenized BCs. It should be noted that opacity is twice as high for the samples obtained by adding the polyelectrolytes to the homogenized BC, which may be due to a greater compactness of the layers as observed in SEM micrographs. Santos et al. [46] showed the same effect for aged BC films.

Gloss deserves special attention. If the samples were obtained by adding polyelectrolytes to the culture medium, the resulting gloss of BC composites were lower compared to the control samples, i.e., a drastic (75%) gloss reduction. This result could be of interest in applications requiring a reduced gloss level or no gloss at all, such as paper restoration. Both sides of samples showed similar gloss values. In contrast, when BTMAXs were added to the homogenized BC, there was no difference in the gloss values when compared with the control samples. In all cases, the a-sides of samples show much higher gloss values than the b-sides. This great difference between sides could be due to the drying method; the side that was in contact with the Petri dishes was much smoother than that which was dried in the air. Gloss values do not depend on whether PS5 or PS6 is used.

The influence of BC structure in the properties of the samples is assessed using Gurley permeance. Gurley porosity values were consistently higher than 900 s, so the closed structure of the BC prevents the air flow through it. This agrees with Yousefi et al. findings [47].

## 4. Conclusions

Incorporation of [4-butyltrimethylammonium]-xylan chloride polyelectrolytes (BTMAXs) on bacterial cellulose (BC) can alter the properties of the cellulose produced, increasing the roughness and the burst index. Gurley porosity values were consistently higher than 900 s, which indicates that the closed structure of the BC prevents the air flow through it. The use of the BTMAXs resulted in materials with less crystallinity index. It also led to a change in the surface free energy, but the behavior depended on the way the biofilms were obtained. The inclusion of BTMAXs in the culture media promotes a remarkable gloss decrease, and the opacity is twice as high in the case of the samples obtained by adding the polyelectrolytes to the homogenized BC. Finally, the described in situ approach proved to be successful for the development of novel nanocomposite materials based on BC and xylan derivatives.

## Figures and Tables

**Table 1:** Crystallinity index (%) of pure BC and composites made by adding [4-butyltrimethylammonium]-xylan chloride polyelectrolytes (BTMAXs) to the culture medium and to the homogenized BC.

	Crystallinity Index (%)
**BC_C_s_**	82.61
**BC_PS5_s_**	75.45
**BC_PS6_s_**	72.86
**BC_C_h_**	80.92
**BC_PS5_h_**	74.48
**BC_PS6_h_**	78.68

**Table 2:** Surface free energy of samples: polar and dispersive components and contact angle with water.

	Surface Free Energy-γ- (mN/m)	Dispersive Component -γ_d_- (mN/m)	Polar Component -γ_p_- (mN/m)	Polar Component/Surface Free Energy -γ_p_/γ- (%)	Contact Angle with Water -α_w_- (°)
**BC_C_s_**	37.7	12.6	25.03	66.5	62.4
**BC_PS5_s_**	24.9	18.2	6.67	26.8	88.6
**BC_PS6_s_**	32.5	16.2	16.28	50.1	73.2
**BC_C_h_**	35.2	14.2	21.00	59.7	65.4
**BC_PS5_h_**	47.7	10.0	37.78	79.1	46.3
**BC_PS6_h_**	42.0	11.8	30.22	71.9	54.0

**Table 3:** Roughness values, obtained with AFM, of pure BC and its composites.

	Rq	
	Side a	Side b
**BC_C_s_**	55.05	52.25
**BC_PS5_s_**	127.00	110.30
**BC_PS6_s_**	102.80	87.80
**BC_C_h_**	84.80	12.77
**BC_PS5_h_**	110.30	38.85
**BC_PS6_h_**	88.10	23.95

**Table 4:** Optical properties of the BC and modified samples with PS_5 and PS_6.

	Yellowness (%)	Opacity (%)	Brightness (%)	Gloss (%)	
				Side a	Side b
**BC_C_s_**	22.46 ± 1.69	23.61 ± 1.50	50.17 ± 1.24	35.26 ± 10.59	40.69 ± 7.74
**BC_PS5_s_**	28.73 ± 0.95	24.25 ± 1.23	41.01 ± 1.05	8.72 ± 2.65	9.93 ± 2.74
**BC_PS6_s_**	18.99 ± 0.97	20.72 ± 1.61	53.19 ± 1.14	10.53 ± 2.19	12.36 ± 2.41
**BC_C_h_**	19.27 ± 1.12	40.64 ± 2.57	56.89 ± 1.01	13.83 ± 0.39	73.83 ± 4.76
**BC_PS5_h_**	25.42 ± 0.78	39.24 ± 4.14	48.79 ± 0.68	14.47 ± 0.33	81.04 ± 4.49
**BC_PS6_h_**	19.03 ± 0.89	40.15 ± 2.22	56.70 ± 0.91	13.58 ± 0.18	79.54 ± 2.31

## References

[1] Iguchi M., Yamanaka S., Budhiono A. (2000). Bacterial cellulose: A masterpiece of nature’s arts. J. Mater. Sci..

[2] Chawla P.R., Bajaj I.B., Survase S.A., Singhal R.S. (2009). Microbial cellulose: Fermentative production and applications. Food Technol. Biotechnol..

[3] Castro C., Zuluaga R., Putaux J.L., Caro G., Mondragon I., Gañán P. (2011). Structural characterization of bacterial cellulose produced by *Gluconacetobacter swingsii* sp. from Colombian agroindustrial wastes. Carbohydr. Polym..

[4] El-Saied H., El-Diwany A.I., Basta A.H., Atwa N.A., El-Ghawas D.E. (2008). Production and characterization of economical bacterial cellulose. Bioresources.

[5] Moon R.J., Martini A., Nairn J., Simonsen J., Youngblood J. (2011). Cellulose nanomaterials review: Structure, properties and nanocomposites. Chem. Soc. Rev..

[6] Moniri M., Moghaddam A.B., Azizi S., Rahim R.A., Ariff A.B., Saad W.Z., Navaderi M., Mohamad R. (2017). Production and Status of Bacterial Cellulose in Biomedical Engineering. Nanomaterials.

[7] Klemm D., Kramer F., Moritz S., Lindstrom T., Ankerfors M., Gray D., Dorris A. (2011). Nanocelluloses: A new family of nature-based materials. Angew. Chem. Int. Ed. Engl..

[8] Bae S., Sugano Y., Shoda M. (2004). Improvement of bacterial cellulose production by addition of agar in a jar fermentor. J. Biosci. Bioeng..

[9] Ishida T., Mitarai M., Sugano Y., Shoda M. (2003). Role of water-soluble polysaccharides in bacterial cellulose production. Biotechnol. Bioeng..

[10] Seifert M., Hesse S., Kabrelian V., Klemm D. (2003). Controlling the water content of never dried and reswollen bacterial cellulose by the addition of water-soluble polymers to the culture medium. J. Polym. Sci. Part A Polym. Chem..

[11] Hirai A., Tsuji M., Yamamoto H., Horii F. (1998). In situ crystallization of bacterial cellulose III. Influence of different polymeric additives on the formation on microfibrils as revealed by transmission electron microscopy. Cellulose.

[12] Yan Z., Chen S., Wang H., Wang B., Wang C., Jiang J. (2008). Cellulose synthesized by *Acetobacter xylinum* in the presence of multi-walled carbon nanotubes. Carbohydr. Res..

[13] Joseph G., Rowe G.E., Margaritis A., Wan W. (2003). Effects of polysaccharide-co-acrylic acid on cellulose production by *Acetobacter xylinum*. J. Chem. Technol. Biotechnol..

[14] Weimer P.J., Hackney J.M., Jung H.J., Hatfield R.D. (1995). Fermentation of bacterial cellulose/xylan composite by mixed ruminal microflora: Implications for the role of polysaccharide matrix interactions in plant cell wall biodegradability. J. Agric. Food Chem..

[15] Whitney S.E.C., Brigham J.E., Darke A.H., Reid J.S.G., Gidley M. (1995). In vitro assembly of cellulose/xyloglucan networks: Ultrastructural and molecular aspects. Plant J..

[16] Tokoh C., Takabe K., Fujita M., Saiki H. (1998). Cellulose synthesized by *Acetobacter xylinum* in the presence of acetyl glucomannan. Cellulose.

[17] Keshk S. (2006). Physical properties of bacterial cellulose sheets produced in presence of lignosulfonate. Enzyme Microb. Technol..

[18] Cheng K.C., Catchmark M.J., Demirci A. (2009). Effect of different additives on bacterial cellulose production by *Acetobacter xylinum* and analysis of material property. Cellulose.

[19] Linder Å., Bergman R., Bodin A., Gatenholm P. (2003). Mechanism of assembly of xylan onto cellulose surfaces. Langmuir.

[20] Köhnke T., Brelid H., Westman G. (2009). Adsorption of cationized barley husk xylan on Kraft pulp fibres: Influence of degree of cationization on adsorption characteristics. Cellulose.

[21] Esker A., Becker U., Jamin S., Beppu S., Renneckar S., Glasser W., Gatenholm P., Tenkanen M. (2002). Self-assembly behavior of some co- and heteropolysaccharides related to hemicelluloses. Hemicelluloses: Science and Technology.

[22] Heinze T., Liebert T., Koschella A. (2006). Esterification of Polysaccharides.

[23] Schwikal K., Heinze T., Saake B., Puls J., Kaya A., Esker A.R. (2011). Properties of spruce sulfite pulp and birch Kraft pulp after sorption of cationic birch xylan. Cellulose.

[24] Vega B., Petzold-Welcke K., Fardim P., Heinze T. (2012). Studies on the fibre surfaces modified with xylan polyelectrolytes. Carbohydr. Polym..

[25] Hestrin S., Schramm M. (1954). Synthesis of cellulose by *Acetobacter xylinum*. 2. Preparation of freeze-dried cells capable of polymerizing glucose to cellulose. Biochem. J..

[26] Santos S.M., Carbajo J.M., Villar J.C. (2013). Bacterial Cellulose from *Gluconacetobacter sucrofermentans* CECT 7291 in the Restoration of Degraded Paper: The Effect of Carbon and Nitrogen Sources on Cellulose Production and Properties. Bioresources.

[27] Retegi A., Gabilondo N., Peña C., Zuluaga R., Castro C., Gañan P., de La Caba K., Mondragon I. (2010). Bacterial cellulose films with controlled microstructure-mechanical property relationship. Cellulose.

[28] Chibowski E. (2003). Surface energy of solid from contact angle hysteresis. Adv. Colloid Interface Sci..

[29] Tiberg F., Daicic J., Fröberg J., Holmberg K. (2001). Surface chemistry of paper. Handbook of Applied Surface and Colloid Chemistry.

[30] Owens D.K., Wendt R.C. (1969). Estimation of the surface free energy of polymers. J. Appl. Polym. Sci..

[31] Tåg C.M., Pykönen M., Rosenholm J.B., Backfolk K. (2009). Wettability of model fountain solutions: The influence on topo-chemical and-physical properties of offset paper. J. Colloid Interface Sci..

[32] Ferreira P.J.T., Moutinho I.M.T., Figueiredo M.M.L. How paper topography affects contact angle measurement. Proceedings of the V Congreso Iberoamericano de Investigación en Celulosa y Papel.

[33] Wenzel R.N. (1936). Resistance of solid surfaces to wetting by water. Ind. Eng. Chem..

[34] Barud H.S., Assuncao M.N., Martines M.A.U., Dexpert-Ghys J., Marques R.F.C., Messaddeq Y., Ribeiro S.J.L. (2008). Bacterial cellulose-silica organic-inorganic hybrids. J. Sol-Gel Sci. Technol..

[35] Huang H.-C., Chen L.-C., Lin S.-B., Hsu C.-P., Chen H.-H. (2010). In situ modification of bacterial cellulose network structure by adding interfering substances during fermentation. Bioresour. Technol..

[36] Khan S., Ul-Islam M., Khattak W.A., Ullah M.W., Park J.K. (2015). Bacterial cellulose-poly(3,4-ethylenedioxythiophene)-poly(styrenesulfonate) composites for optoelectronic applications. Carbohydr. Polym..

[37] Kim S.H., Lee C.M., Kafle K. (2013). Characterization of crystalline cellulose in biomass: Basic principles, applications, and limitations of XRD, NMR, IR, Raman, and SFG. Korean J. Chem. Eng..

[38] Ul-Islam M., Shah N., Ha J.H., Park J.K. (2011). Effect of chitosan penetration on physico-chemical and mechanical properties of bacterial cellulose. Korean J. Chem. Eng..

[39] Yano S., Maeda H., Nakajima M., Hagiwarta T., Sawaguchi T. (2008). Preparation and mechanical properties of bacterial cellulose nanocomposites loaded with silica nanoparticles. Cellulose.

[40] Lopes T.D., Riegel-Vidotti I.C., Grein A., Tischer C.A., Faria-Tischer P.C.S. (2014). Bacterial cellulose and hyaluronic acid hybrid membranes: Production and characterization. Int. J. Biol. Macromol..

[41] Harnett E.M., Alderman J., Wood T. (2007). The surface energy of various biomaterials coated with adhesion molecules used in cell culture. Colloids Surf. B Biointerfaces.

[42] Oliveira P., Conceição S., Santos N.F., Velho J., Ferreira P. The influence on rheological modifiers on coated papers: A comparision between CMC and MHPC. Proceedings of the III Congreso Iberoamericano de Investigación en Celulosa y Papel.

[43] Moutinho I.M.T., Ferreira P.J.T., Figueiredo M.L. (2007). Impact of surface sizing on inkjet printing quality. Ind. Eng. Chem. Res..

[44] Yamamoto H., Horii F., Hirai A. (1996). In-situ crystallization of bacterial cellulose II. Influences of polymeric additives with different molecular weights on the formation of Celluloses I_α_ and I_β_ at the early stage of incubation. Cellulose.

[45] Yamamoto H., Horii F. (1994). In Situ crystallization of bacterial cellulose I. Influences of polymeric additives, stirring and temperature on the formation celluloses I_α_ and I_β_ as revealed by cross polarization/magic angle spinning (CP/MAS) ^13^C NMR spectroscopy. Cellulose.

[46] Santos S.M., Carbajo J.M., Quintana E., Ibarra D., Gómez N., Ladero M., Eugenio M.E., Villar J.C. (2015). Characterization of purified bacterial cellulose focused on its use on paper restoration. Carbohydr. Polym..

[47] Yousefi H., Faezipour S., Hedjazi S., Mousavi M.M., Azusa Y., Heidaria A.H. (2013). Comparative study of paper and nanopaper properties prepared from bacterial cellulose nanofibers and fibres/ground cellulose nanofibers of canola straw. Ind. Crops Prod..

